# Evaluation of the Fairness of Urban Lakes’ Distribution Based on Spatialization of Population Data: A Case Study of Wuhan Urban Development Zone

**DOI:** 10.3390/ijerph16244994

**Published:** 2019-12-08

**Authors:** Jing Wu, Shen Yang, Xu Zhang

**Affiliations:** School of Urban Design, Wuhan University, Wuhan 430072, China; 2016301120042@whu.edu.cn (S.Y.); 2016301530004@whu.edu.cn (X.Z.)

**Keywords:** fairness of lake distribution, population data spatialization, accessibility, Wuhan City

## Abstract

Lake reclamation for urban construction has caused serious damage to lakes in cities undergoing rapid urbanization. This process affects urban ecological environment and leads to inconsistent urban expansion, population surge, and uneven distribution of urban lakes. This study measured the fairness of urban lakes’ distribution and explored the spatial matching relationship between service supply and user group demand. The interpretation and analysis of Wuhan’s remote sensing images, population, administrative area, traffic network, and other data in 2018 were used as the basis. Specifically, the spatial distribution pattern and fairness of lakes’ distribution in Wuhan urban development zone were investigated. This study establishes a geographic weighted regression (GWR) model of land cover types and population data based on a spatialization method of population data based on land use, and uses population spatial data and network accessibility analysis results to evaluate lake service levels in the study area. Macroscopically, the correlation analysis of sequence variables and Gini coefficient analysis method are used to measure the fairness of the Wuhan lake distribution problem and equilibrium degree, and the location entropy analysis is used to quantitatively analyze the fairness of lakes and Wuhan streets from the perspective of supply and demand location entropy. Levels improve the accuracy of the research. Results showed that (1) the area covered by lakes in Wuhan urban development zone is 1007.96 km^2^ within 60-min of walking, accounting for 30.6% of the total area of the study area. This area can house 5,050,275 people, accounting for 60.8% of Wuhan’s total population. (2) The lakes in the central city area are less fair than the lakes outside the Third Ring Road. (3) The service level of North Lake is the highest among all the lakes in the study area, and that of Hou Lake is the lowest. (4) The spatial layout of the fairness of the lakes’ distribution is roughly distributed in circles. The fairness level collapses toward the city center, indicating that the closer to the city center, the lower the fairness level.

## 1. Introduction

Water has been widely used since early human civilization as a necessary source of survival, irrigation, and transportation. Many famous cities in the world are located at the intersection of large rivers or water and land [1], and Wuhan is a typical example. As an important element in physics, the aesthetic landscape, and the environment, water is vital to environmental psychology, landscape design, and tourism research [2]. Existing studies on urban water focus on water recycling [3], environmental pollution from heavy rains and floods [4], environmental health risks of water supply sources [5], and water-sensitive urban design [6]. In the contexts of economics, health, and the landscape environment, few studies on the fairness of urban waters’ distribution are conducted in the fields of sociology and big data. As an important part of urban water space, urban lakeside space can provide various ecosystem services, such as management of rainwater [7], alleviation of the heat island effect [8], and regulation of the urban climate. Urban water space offers many social and economic benefits, such as providing ecosystem services to meet the needs of urban residents for relaxing and contacting with nature, manipulating housing prices [9] to enhance the value of neighboring areas, and affecting residents’ health and emotional perception [10]. Therefore, rationally improving the accessibility and per capita equity of urban lakes is crucial to enhancing the overall well-being of urban residents. Doing so can also enable women, children, the elderly, low-income groups, and other vulnerable groups to reap the benefits of resource acquisition and social development [11]. With the acceleration of urbanization, urban water environment resources cannot meet the needs of rapidly expanding populations in many cities, and the uneven distribution of water environment resources has become increasingly prominent [12,13].

The research on social fairness and justice focuses on economic fairness, social fairness, and environmental justice [14,15]. Economic equity, economic justice, and urban political economics are investigated by urban research scholars, such as David Harvey, John Logan and Harvey Molotch, and Susan Fainstein [16]. Social justice reflects the social differences, including democracy and transcending economic distribution [17,18]. The concept of social justice includes historical issues, such as site ownership, actual availability, and local discrimination. Social equity should also pay attention to urban lakeside areas, which is also called the “social production of space” by cultural geographers [19]. Environmental justice refers to the distribution of public green space within the walking distance of high-income groups, and has relatively low contact with low-income people [20]. The classical perspective of environmental justice is extended to cities’ lakeside areas [21]. This study evaluates the fairness of urban lakes’ distribution on the basis of population data spatialization to serve as reference for urban lakeside and blue–green space construction.

A city’s lake district is a special and valuable type of public space with shared environmental and public advantages that are crucial to sustainable urban development. The reconstruction of urban lakeside areas is mainly challenged by the need to build a place for resisting established local and regional injustices rather than the technical, financial, and administrative aspects [22,23]. The fairness of public services should be assessed on the basis of the spatial and quantitative relationships between user needs and object supply.

This study assesses the fairness of urban lakes’ distribution at a finer spatial scale through population data spatialization and explores the fair distribution of lakes in terms of equal access to lake resources by different social groups. A spatial distribution map depicting the distribution of the population of the study area with high precision was obtained, and the corresponding population demand was quantified by constructing a regression model of the land cover types and statistical population. The accessibility of lakes was quantitatively evaluated using ArcGIS network analysis tools. Location entropy analysis was performed to assess the fairness of lakes’ distribution in the study area and classify them spatially. This study aimed to provide a reference for the planning and decision-making of urban planners and to promote social equity.

## 2. Research Areas and Data Sources

### 2.1. Research Area

Wuhan is located at 113°41′–115°05′ east longitude and 22°29′–31°58′ north latitude in the World Geodetic System 1984. It is located in the eastern part of the Jianghan Plain, middle reaches of the Yangtze River, and the center of China’s hinterland. Wuhan is the capital of Hubei Province. It is the central city of “1 + 8 City Circle” and a supporting experimental area for China’s resource-saving and environment-friendly society construction, with a total area of 8494.41 km^2^ [24].

Wuhan is rich in water resources, accounting for a quarter of the total area of the city. A total of 140 rivers with a length of more than 5 km are found in the study area. The third longest river in the world, the Yangtze River, and the largest tributary of the Yangtze River, the Han River, meet at the center of the city, forming a unique cross-strait of three districts and a unique urban spatial pattern in Wuhan. Wuhan has 273 reservoirs and 166 lakes, in which 56 of them are located in urban development areas. Unsurprisingly, Wuhan is also known as the “city of thousands of lakes” [25].

**Figure 1:**
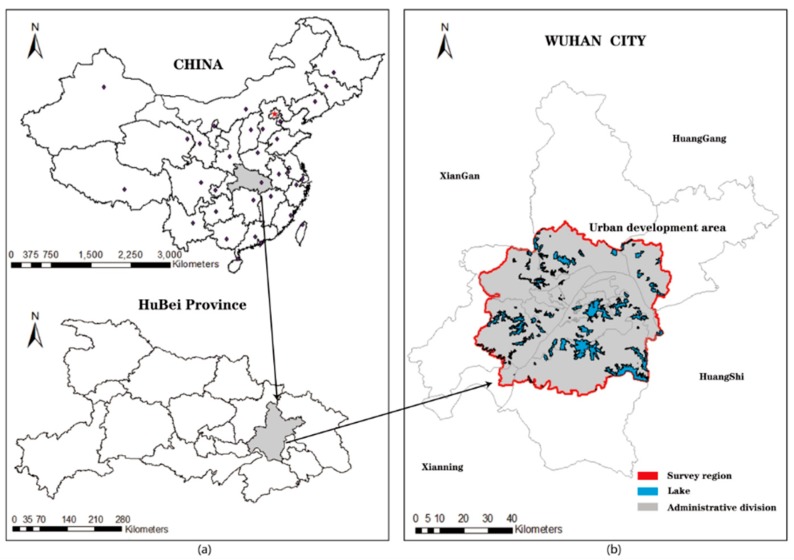
Research area: (**a**) location of Wuhan City (**b**) general location of the urban development area in Wuhan and spatial layout of the lakes. The distribution of lakes is relatively scattered. Large lakes are located in the southeast of the research area.

Wuhan consists of 13 administrative districts (including seven central urban areas and five surrounding urban areas) and four development zones. The scope of this study is Wuhan Urban Development Zone, as shown in Figure 1, including Jiang’an, Jianghan, Qikou, Hanyang, Wuchang, Hongshan, Qingshan, Caidian, Hannan, Jiangxia, Huangpi, and Xinzhou Districts, which are part of the East and West Lake Districts. The study area has a total of 148 streets as shown in Table 1, with a total area of 3294.31 km^2^ (including lakes in the region of 309.39 km^2^). The urban construction land in the urban development zone will reach 802 km^2^, the urban population will reach 8.8 million, and the per capita urban construction land will reach 91.1 m^2^ by 2020 [26].

The 26th meeting of the Standing Committee of the 13th People’s Congress of Wuhan on 9 January 2015 and the 14th meeting of the Standing Committee of the 12th People’s Congress of Hubei Province on 1 April 2015 approved the Wuhan Lake protection regulations. This is an environmental regulation formulated by Wuhan to strengthen the protection of lakes in this city, prevent lakes from being occupied and polluted, and for the maintenance of the ecological environment. Wuhan has 166 lakes, in which 40 of them are located in the central city, as shown in Table 2, where Tangsong Lake (47.6 km^2^) is the largest urban lake in China [27]. Many large lakes are located on either side of the two main rivers, forming a network of lakes. The lake distribution generally presents a spatial pattern of “large mixed habitats and small settlements”, that is, the overall distribution is relatively scattered. The concentrations are in the central and southern parts of Wuhan, but clusters are noted around East, Tangxun, and Taizi Lakes. Lakes in the central city are generally smaller, whereas the lakes in the suburbs are larger [28].

### 2.2. Data Source and Preprocessing

#### 2.2.1. Population Vectorization

**Figure 2:**
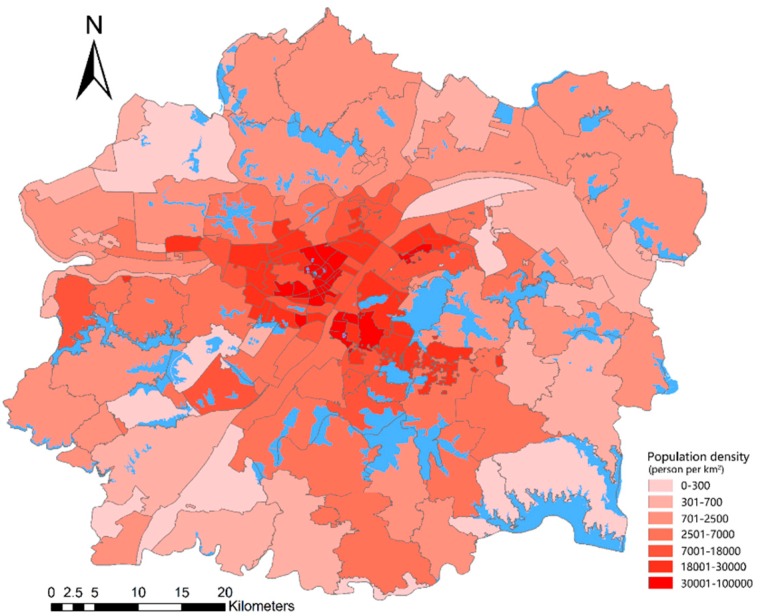
Street population density in Wuhan urban development area in 2010.

The population data is the spatial data of the 2018 statistical yearbook census in Wuhan and the corresponding population density distribution map is generated on the basis of administrative division data, as shown in Figure 2. These data are based on the vectorization of the grid map of each street. The densely populated areas are mainly concentrated in the central area of the urban development zone. The closer to the edge of the urban development zone, the lower the population density.

#### 2.2.2. Study Lake and Road Network Extraction

The lake vector data for 2018 used in this study were derived from the remote sensing images of Landsat 8. A water form method was used to extract the lake shape data, and the extracted lake results were evaluated and corrected through manual visual interpretation to improve the accuracy of lake extraction.

**Figure 3:**
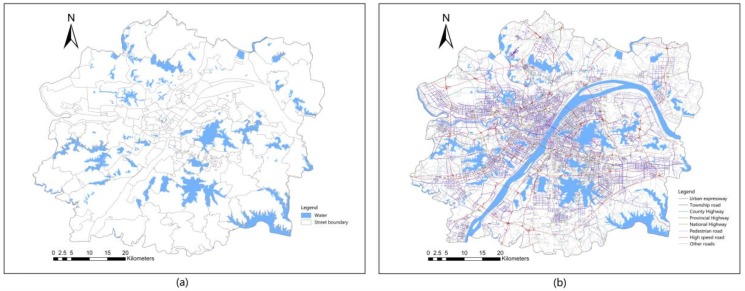
(**a**) Wuhan urban development area lakes and their names; (**b**) road network.

Road vector data for 2018 were provided by Wuhan Planning and Design Institute, as shown in Figure 3. The vector road network dataset of the study area was constructed after correcting the topology errors.

#### 2.2.3. Acquisition of Land Cover Types in the Study Area

The land cover data were obtained from the interpretation of Landsat 8 satellite remote sensing images on 8 April 2018. Sensor: OLI (A sensor for obtaining remote sensing data), resolution: 30/15 M, data recognition: LC81230392018098LGN0, cloud amount (%): 8.9%. The images were downloaded from the China Geospatial Data Cloud website (http://www.gscloud.cn/).

Urban lands are frequently defined as nonflat areas dominated by man-made surfaces, such as roads and buildings. This study focused on residential, forest, industrial, and road-based land use classifications [29].

First, the images were preprocessed (radiation calibration, atmospheric correction, blending, cutting, etc.) on Envi5.3 software (ITT Visual Information Solutions, New York, NY, USA) and were fused by combining different digital images to create new images using the system’s own algorithms. Image fusion combines spatial and spectral data from multiple images to exploit all the available information. It was implemented to fully utilize the spatial information of the remote sensing images (30 m) [30].

Then, Albers equal-area conic projection was used as the reprojected coordinate system, and all the images were resampled to a 15 m × 15 m resolution, resulting in a small drawing unit or small urban land area of 225 m^2^.

**Figure 4:**
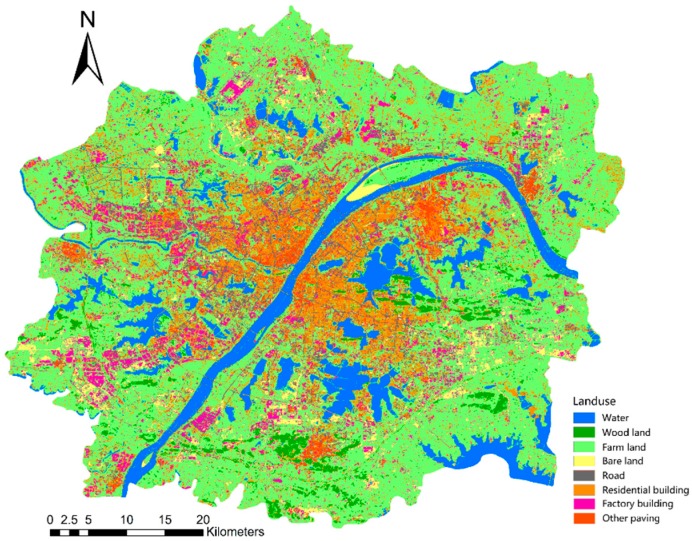
Land use map of Wuhan urban development area.

Finally, the images were classified using a supervised maximum likelihood classification. The land cover types were divided into eight categories, namely, water bodies, woodlands, farmlands, bare lands, roads, residential lands, industrial lands, and other paving, the classification image is shown in Figure 4. Maximum likelihood estimation assumes that each type of statistic for each band is normally distributed, calculates the likelihood that a given pixel belongs to a coverage class corresponding to a training sample, and the pixels are assigned to the class with the greatest likelihood.

Land cover type training and validation samples were extracted from Google Earth and Landsat images by visual interpretation. We selected 10 regions of interest for each type of land cover as evaluation samples and used a confusion matrix to estimate the classification accuracy. The results showed an overall accuracy of 82%.

## 3. Method

### 3.1. Population Data Spatialization

#### 3.1.1. Spatial Analysis of Population Data Based on Land Cover

The analysis assumed that the population density is the same for the same type of land cover. The land cover type data were obtained using remote sensing images, the land cover type data unit was used as the explanatory variable, and the demographic data were used as the dependent variable to construct a multivariate regression model. The formula is expressed as follows:

(1) $$P_{i}=\sum_{j=1}^{n}a_{j}\times s_{\mathrm{ij}}+b,$$

where *p_i_* is the statistical population of the *i*th block in the study area, *a_j_* is the population distribution coefficient under the *j* type land cover type, S_ij_ is the area of the *j* land cover type in the *i* block, and *n* represents the number of land covertypes. According to the principle of “no land without population”, the constant b is 0.

As shown in Equation (1), we simulated the predicted population value of each block and evaluated the error using the following equation:

(2) $$E=\frac{p_{i}^{\prime }-p_{i}}{p_{i}}\times 100\%,$$

where *E* is the relative error, *p_i_* is the actual population of block *i* in the study area, and *p’_i_* is the predicted population in block *i*.

Considering that the model assumes that the population distribution coefficients of the same land cover type are the same, a deviation is noted between the predicted and the actual population. To ensure that the sum of the population data in the grid is consistent with the actual population, the initial coefficient was corrected and recalibrated using the following equation:

(3) $$a_{ij}^{\prime }=\frac{p_{i}}{p_{i}^{\prime }}\times a_{j},$$

where *α’_ij_* is the population distribution coefficient under the category *j* land cover type of corrected block *i*, *p_i_* is the actual population of block *i* in the study area, and *p’_i_* is the predicted population in block *i*.

A grid of a certain size was constructed, and the land cover area in the grid was calculated. The population in the calculation grid was inverted on the basis of the correction coefficient to realize the rasterization of the street population data. The grid population data were calculated using the following equation:

(4) $$pop_{ik}=\sum_{j=1}^{n}\alpha_{ij}^{\prime }\times S_{ijk},$$

where *pop_ik_* is the Culation of the *k*th grid unit in block *i*, *α’_ij_* is the population distribution coefficient under the category *j* land cover type of corrected block *i*, and *S_ijk_* is the area of the *j*th land cover type on the *k* grid units in block *i*.

#### 3.1.2. Geographically Weighted Regression (GWR)

In geospatial analysis, the observed values of variables are generally obtained as sample units based on a given geographic unit. The relationship of variables changes when the change of geographical location is adjusted on the basis of geographical location. The change is called the spatial nonstationary adjustment. Fotheringham proposed a GWR model based on the previous research on local regression and variable parameters to solve the distortion of the analysis results caused by the roughness of spatial data [31,32]. The GWR model aims to increase the distance function as a weight using the ordinary least squares (OLS) regression model to deal with the spatial instability of the data, and the correlation coefficient changes with the position. The GWR model extends the traditional regression framework by allowing local parameter estimation rather than global parameter estimation. The spatial characteristics of the data are incorporated into the model for regression analysis by assuming that the regression coefficient is a positional function of the geographic location of the observation point in the linear regression model. The spatial characteristics of the relationship provide the conditions. The model is expressed as follows:

(5) $$\gamma_{i}=\beta_{0}(u_{i},v_{i})+\sum_{k=1}^{p}\beta_{k}(u_{i},v_{i})x_{ik}+\varepsilon_{i},$$

where (*γ_i_*; *x_i_*_1_, *x_i_*_2_,..., *x_ip_*) denotes geographical location (*u_i_, v_i_*) at dependent variable *γ* with independent variables *x*_1_, *x*_2_,..., *x_p_* observations (*i* = 1, 2,…, *n*), and β_k_(*u_i_, v_i_*) (*k* = 0, 1,..., *p*) is the unknown parameter at observation point (*u_i_*, *v_i_*), which is an unknown function of (*u_i_*, *v_i_*). *ε_i_*(*i* = 1, 2,..., *n*) is independent and identically distributed, and the random error is usually assumed to obey *N*(0, σ2).

In this study, we used the demographic data as a dependent variable and the land cover area as an independent variable on ArcGIS10.1 GWR (Environmental Systems Research Institute, RL, CA, USA). An adaptive method was used to calibrate the weighting function, and the optimal bandwidth was determined using the minimum Akaike information criterion (AIC).

The performance of OLS and GWR models is usually compared in two aspects, namely, the prediction ability and the spatial autocorrelation of processed variables [33,34]. We used a comparison model of adjusted R^2^ and AIC. Adjusted R^2^ is a measure of fitness, and the higher the adjusted R^2^ is, the stronger the explanatory ability of the variable is. AIC is a measure of model performance and helps to compare different regression models. The model with a smaller AIC value is considered to be better when the difference between the two models is greater than three. Global Moran’s I index was calculated based on the spatial autocorrelation. Moran’s I value ranges from −1 to 1, and values close to 0 indicate small or no spatial autocorrelation. A normal Moran’s I index value indicates an aggregation trend, whereas a negative Moran’s I index value indicates a dispersion trend. The assumption that the residual follows a random distribution is violated when the distribution of residuals obtained from the regression model has significant spatial autocorrelation. This condition indicates that the results of the model are unreliable.

### 3.2. Network Reachability Analysis

Network accessibility analysis calculates the service range of a facility at a certain cost based on the road network of a particular travel mode (walking, cycling, taking the bus, or driving a private car). Network analysis can provide more accurate measurements than simple buffer methods because the former considers actual travel routes and distances [35,36]. A basic network mainly includes centers, links, nodes, and costs.

Previous studies on the accessibility of urban networks focus on the accessibility of urban parks. They used the real entrances and exits of urban park greens as accessible points, with urban roads as the link, and nodes as the intersections of roads. Cost was expressed in terms of road time [37,38]. No actual entrance and exit were found because the lake boundary is long and narrow. For convenience, this study established a 100-m buffer along the lake boundary. The intersection of the peripheral boundary of the buffer and the road network was defined as the accessible point of the lake.

This study used the time cost of residents to walk to the lake’s accessible points as a quantitative measure of accessibility. The time cost of each link could be calculated based on the road length, with 1 m/s as the average walking speed, and the time cost of the road intersection was set to 30 s. The radiation range of the lake along the road was obtained using four gradients (15, 30, 45, and 60 min) to determine the maximum service level of the lake.

### 3.3. Sample Data and Normalization of the Processed Data 

Normalization is the preprocessing stage for many types of problems. In particular, normalization in geospatial and urban planning data analysis and processing plays an important role in manipulating the data before narrowing the data range or expanding the data range before the data are used in subsequent stages.

Data standardization (normalization) is a fundamental pre-processing stage of data mining. Values of different parameters used in the analysis can be expressed in different units or intervals, thus they must be made compatible by their normalization in the 0–1 range before their use. Convenient data processing should be standardized by mapping the data within the 0–1 range to solve the comparability between data indicators. After the original data are processed through data standardization, each indicator has the same order of magnitude, which is suitable for comprehensive comparative evaluation.

The evaluated indicators are divided into positive, inverse, and appropriate indicators. A positive index indicates that the higher the index value is, the better the evaluated object is. The lower the index is, the worse the evaluated object is. An appropriate index indicates that the closer the number of indicators is to a certain value, the better the evaluated object is.

This study used positive indicators, and the normalization equation was expressed as follows:

(6) $$x_{i}^{\prime }=\frac{x_{i}-x_{\min}}{x_{\max}-x_{\min}},$$

where *x_i_*, *x’_i_* represent the values before and after data normalization, respectively, and *x*_min_ and *x*_max_ represent the minimum and maximum values in the sample data, respectively.

### 3.4. Evaluation of Lake Service Level

The perimeter of the lake boundary determines its service possibilities to a certain extent. The longer the perimeter of the lake is, the larger the lakeside space will be, and the larger the activity space will be, and the population around the lake quantifies the lake service capacity and service benefits. In this study, the population covered by lakes in the 15, 30, 45, and 60 min service areas were selected as research objects. The service level of lakes was evaluated based on the number of people served per unit length of the lake boundary. The equation was expressed as follows:

(7) $$R=\frac{N}{L},$$

where *R* is the number of service populations per unit length of the lake, *N* is the number of people covered in different service areas (15, 30, 45, and 60 min), and *L* is the perimeter of the lake.

### 3.5. Quantitative Evaluation of the Fairness of Lakes Distribution 

#### 3.5.1. Demand Index Calculation

According to previous research on the neighborhood of urban and rural planning, the urban lakeside water space layout based on population demand can be considered fair. In many previous studies on urban public resource allocation, such as park green space, schools, and hospitals, the demand index is frequently used to evaluate the fairness of resource distribution [39,40,41]. The demand for public resources, such as lakeside space, is frequently related to indicators, such as gender, age, social status, and income. Therefore, research should focus on vulnerable groups in the city to assess social fairness. This study used the relevant research on the definition and classification basis of vulnerable groups [42], combined with the actual needs of urban waterfront space of women, children, the elderly, and migrants as the main body of urban lakeside space demand. The total population density, female population density, youth population density (those who are 0–14 years old), elderly population density (those over 65 years old), and non-native population density were selected based on the 2018 statistical yearbook census in Wuhan with consideration of the differences in the location and size of each street. Other indicators were used to characterize the degree of residents’ demand for urban lakes. After the normalization of the abovementioned various types of population index data using Equation (6), the total population density, female population density, youth population density, elderly population density, and non-native population density were given with weights of 0.2, 0.1, 0.25, 0.25, and 0.2 in terms of the degree of demand of different populations for urban lakes. The results were normalized again after the weighted summation, and the demand index of lakes for each street resident was obtained:

(8) $$R_{i}=\sum_{i=1}^{n}\sum_{j=1}^{5}X_{ij}\times b_{j},$$

where *R_i_* is the lake demand index of the *i*th street, *X_ij_* is the value of the *j*th index of the *i*th street, and *b_j_* is the weight of the *j*th-type index.

#### 3.5.2. Measurement of Supply Level

The proportion of the population of each street in the 60-min reach of the lake to the total population of each street was selected based on the results of population spatialization and network analysis to evaluate the supply level of each street lake. After the data normalization using Equation (6), the supply level of the lake was divided into five levels using quantile classification, namely, extremely low, low, medium, high, and extremely high.

#### 3.5.3. Correlation Analysis of Sequencing Variables

The quantitative analysis of fairness was conducted via the correlation analysis of sequence variables to quantitatively evaluate the fairness of lakes’ distribution under the street scale of the study area. The correlation analysis of sequenced variables reflects the order relationship between the two variables by statistically analyzing the correlation of the ranks after the two variables are sorted [43]. This study used the Spearman rank correlation and Kensington rank correlation coefficients to perform linear correlation analysis on the street scale demand index and supply level on SPSS for quantitatively measuring the fairness of lakes’ distribution at the street scale, and a two-sided test was adopted. SPSS is a software product for statistical analysis, data mining, predictive analysis, and decision support tasks. The correlation coefficient was expressed as follows:

(9) $$R=1-\frac{6\sum_{i=1}^{n}{\left(U_{i}-V_{i}\right)}^{2}}{n(n^{2}-1)},$$

where *R* is the Spearman rank correlation coefficient; *U* and *V* are the ranks of the two variables, respectively; and *n* is the sample size:

(10) $$T=1-\frac{4K}{n(n-1)},$$

where *T* is the Kensington rank correlation coefficient, *K* is the number of nonuniform pairs obtained from the rank data of the variable, and *n* is the sample size.

#### 3.5.4. Analysis of Gini Coefficient

The Gini coefficient was proposed by the Italian economist Corrado Gini in 1912 and is an analytical indicator used internationally to comprehensively assess the income distribution gap of residents [44]. Its value is between 0 and 1; the lower the value of the Gini coefficient, the more uniform the distribution of social wealth. The Gini coefficient defines the threshold of the difference between the rich and the poor. It can objectively and intuitively present the gap between the rich and the poor. It has certain reference significance for forecasting, early warning, and preventing the gap between the rich and the poor. For the division of the Gini coefficient score segment, the relevant UN organization regulations were used, as shown in Table 3.

The Gini coefficient can be used not only for the study of income distribution problems but also for many analyses of distribution problems and equilibrium, covering multi-domain and multi-level applications [45]. This paper aimed to analyze the fairness of urban lake distribution. Therefore, the Gini coefficient analysis method was used to evaluate the fairness of lakes’ distribution from a macro perspective. The equation for calculating the Gini coefficient was:

(11) $$G=1-\sum_{k=1}^{n}\left(D_{k}-D_{k-1}\right)(Q_{k}+Q_{k-1}),$$

where: *D_k_* is the cumulative ratio of the lake demand level of the *k*th street, *k* = 1, 2, …, *n*, *D*_0_ = 0, *D_n_* = 1; *Q_k_* is the cumulative ratio of the lake supply level of the *k*th street, *k* = 1, 2, …, *n*, *Q*_0_ = 0, *Q_n_* = 1.

#### 3.5.5. Location Entropy Analysis

Location entropy was first proposed by P. Haggett and applied to spatial location studies. The correlation analysis of the ordering variables and the Gini coefficient only expresses the supply and demand balance of urban lakes on a macroscopic level, and cannot express the spatial matching of the supply and demand of the lake. Using the location entropy method to express the spatial pattern of the balance of supply and demand in the lake, the evaluation scale can be narrowed down to the street space. In this paper, the location entropy of each street was the ratio of the supply:demand ratio of lakes in the street to the supply:demand ratio of lakes in the whole study area. The Equation was as follows:

(12) $$LQ_{i}=(Q_{i}/D_{i})(Q/D),$$

Among them, *LQ_i_* is the location entropy of *i* street, *Q_i_* is the lake supply level in *i* street, *D_i_* is the lake demand level in *i* street, *Q* is the total supply level of lake in the study area, and *D* is the overall level of demand for lakes in the region. If the location entropy of the street is greater than 1, it indicates that the lake supply and demand balance value of the street is higher than the overall level of the lake supply and demand balance in the study area; if the street location entropy is less than 1, it indicates that the lake supply and demand balance value of the street is lower than the lake in the study area.

## 4. Results

### 4.1. Spatial Population Data Based on Land Cover Types

#### 4.1.1. Performance Comparison of the OLS and GWR Models

A close relationship is observed between the spatial distribution of the population and type of land cover. We used Landsat8 remote sensing image data to classify the land cover types in the Wuhan urban development zone into nine categories, namely, water bodies, woodlands, farmland, bare land, roads, residential land, industrial land, other paving, and unclassified land. The area for each land cover type was counted as a variable parameter by street unit, and Spearman correlation analysis was performed using the total population data of each street to establish a regression model. The results in Table 4 indicate a significant correlation among population distribution and roads, residential land, other paving, and unclassified land.

The OLS and GWR models were constructed by combining various land cover types as explanatory variables and using the total population of each street as the dependent variable. Finally, we find that when roads, residential land, and other paving are used as explanatory variables, the fitting optimization of the model is optimal.

**Figure 5:**
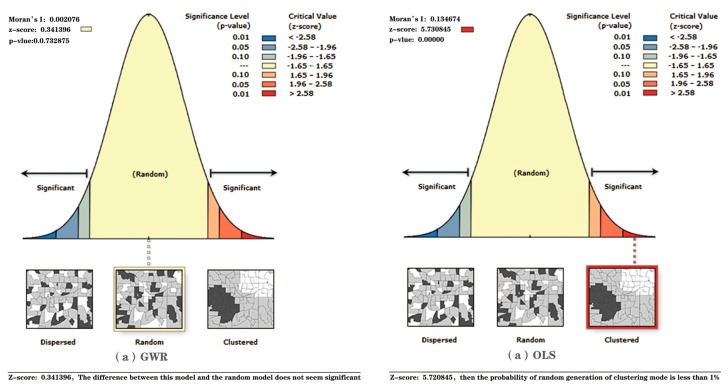
Comparison of residual Moran’s I index: (**a**) Geographically Weighted Regression model; (**b**) Ordinary Least Squares model.

Table 5 shows the variance inflation factors (VIF) of the three land cover types, where VIF (road) = 5.21, VIF (residential land) = 4.32, and VIF (other paving) = 1.55, all of which are less than 7.5. This finding indicates that no multicollinearity exists between the variables. The AIC values of the OLS and GWR models are 3883.671 and 3813.341, respectively. The adjusted R^2^ value of the GWR model is 0.549, which is higher than 0.180 of the OLS model. Figure 5 shows that Moran’s I is 0.135, z score is 5.78, and *p*-value is 0.000 for the OLS model, which indicate that the standardized residual distribution of the OLS model shows a clear clustering pattern with a 95% confidence level. Conversely, Moran’s I is 0.002, z score is 0.341, and *p*-value is 0.732 for the GWR model. Thus, the standardized residual distribution of the GWR model is a random pattern. The abovementioned factors indicate that the GWR model is superior to the OLS model in all aspects.

#### 4.1.2. Implementation of Population Data Spatialization

**Figure 6:**
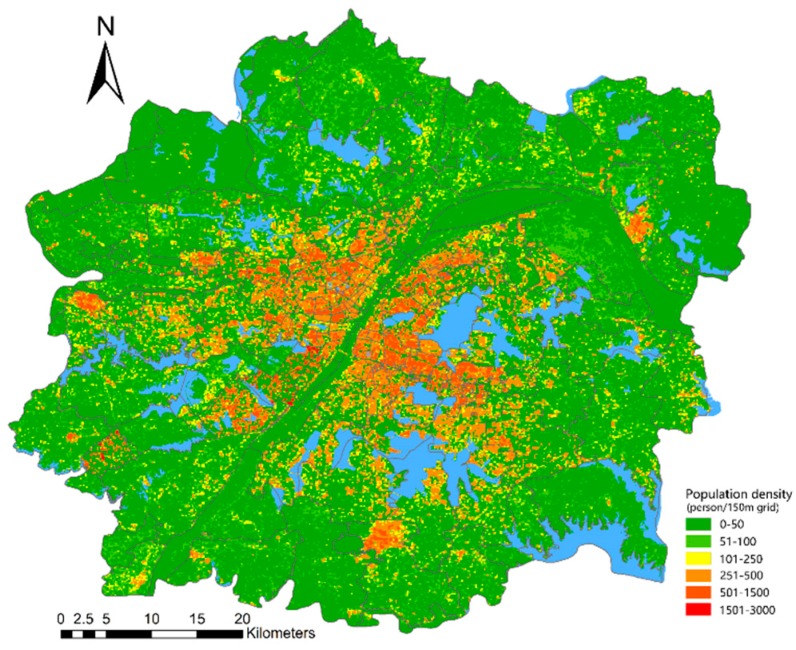
Map of the spatialized population density of Wuhan urban development zone in 2018.

According to Equation (2), the test model simulates the overall error of population spatialization. The total simulated population is 8,523,371, whereas the actual population is 8,308,841. Thus, the overall relative error is 2.58%. The average relative error of spatial simulation of the simulation population for each street is 40.37%. Therefore, correcting the error is necessary. To ensure that the sum of the total data in the grid is consistent with the actual total number, we used Equation (3). The initial coefficient was corrected to obtain a raster image of the corrected regression coefficient. The study considered the population density and street scale and set the grid size to 150 m. Furthermore, a fishing net tool was used to obtain a grid image of the Wuhan urban development zone, and the areas for roads and residential land in each grid were calculated. Using Equation (4), grid and street vectors allocated the population to each grid to achieve spatialization of the population data, as shown in Figure 6.

### 4.2. Urban Lake Accessibility Analysis

#### 4.2.1. Lake Accessibility Gradient Analysis

**Figure 7:**
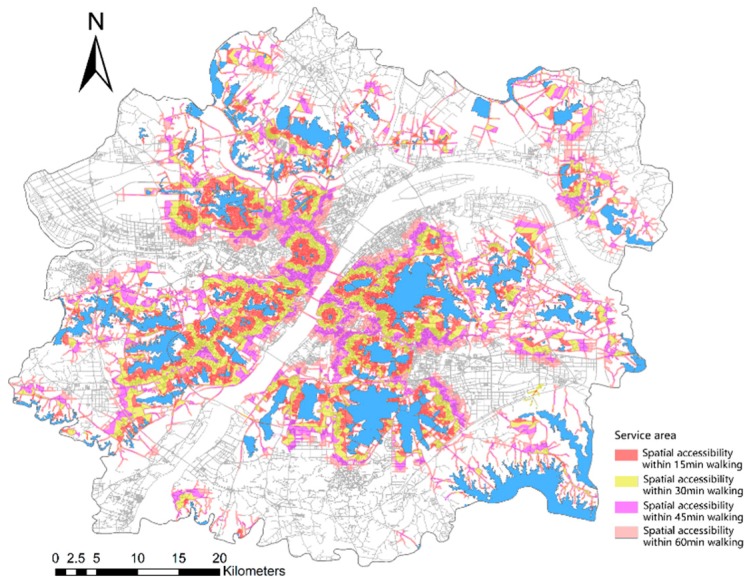
Service areas of lakes according to the network analysis methods.

According to the network analysis of the accessibility of urban lakes, the service areas of the lakes were 15, 30, 45, and 60 min. Figure 7 depicts the results. Areas with good accessibility are mainly concentrated in the East, Shahu, Nanhu, Tangxun, and Huangjia Lakes in Wuchang; Jinyin, Huangtan, Taizi, and North Lakes in Hankou; and Moon, Ink, Longyang, Sanjiao, Houguan, Nantaizi, and Zhushan Lakes in Hanyang. Areas with poor accessibility are mainly located at the edge of the study area. Few lakes can be found in these areas, or the road transportation system around the lake is underdeveloped, such as Niushan and Liangzihou Lakes.

**Figure 8:**
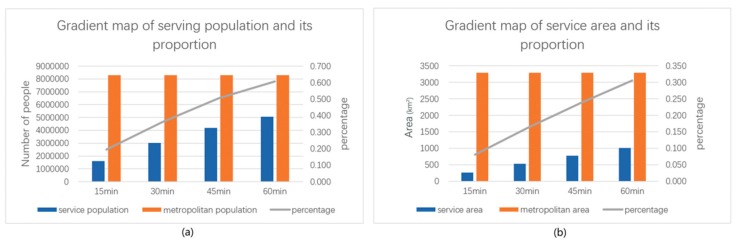
Population and area statistics in the lake service area: (**a**) demographic chart; (**b**) area chart.

Figure 8 and Table 6 provide the available statistics. The coverage area of the lake within 15-min of walking reaches 267.78 km^2^, which accounts for 8.13% of the total area, covers a population of 1,615,420, and represents 19.44% of the total population of the study area. Within 30-min of walking, the coverage area reaches 532.53 km^2^, which accounts for 16.17% of the total area, covers a population of 3,014,743, and represents 36.28% of the total population of the study area. After 45-min of walking, the coverage area spans 777.98 km^2^, which covers 23.62% of the total area, a population of 4,193,591, and 50.47% of the total population of the study area. After 60-min of walking, the coverage area reaches 1007.97 km^2^, which represents 30.60% of the total area, a population of 5,050,274, and 60.78% of the total population of the study area.

In terms of urban spatial structure, areas with high accessibility are mainly concentrated in urban centers, such as the junction between Jianghan and Jiang’an Districts, Wuchang District, Hanyang District, and Qingshan District, and the majority of the streets along the Yangtze River. The green areas in these locations are not only concentrated, but the road facilities are relatively complete.

By comparing the coverage area and population difference within the gradient service areas of the lake, we find that the proportion of the coverage population is approximately twice that of the coverage area. This result indicates that people tend to live around the lake in the urban development zone.

#### 4.2.2. Overlapping Analysis of Lake Accessibility and Population Spatialization

**Figure 9:**
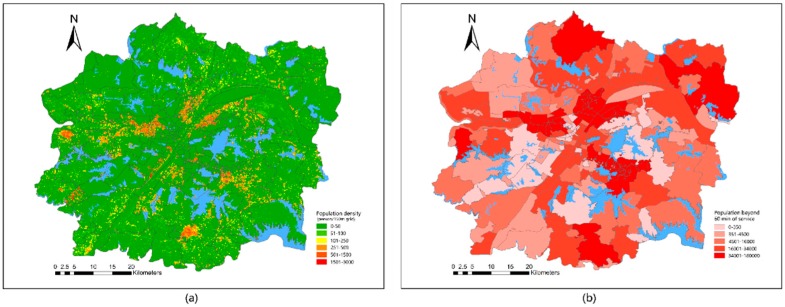
Spatialized population density map: (**a**) spatial distribution of low-accessibility population; (**b**) low-accessibility population at the street scale.

Following the analysis of spatial distribution of the population and network accessibility, spatial superposition analysis was used to obtain results with low-reachability spatial distribution (Figure 9a). After the 60-min walk, the most densely populated streets are Gutian, Hanjiadang, and Zongguan in the middle of Jianghan District; Erqi, Xincun, and Danchi in the middle of Jiang’an District; Hongweilu, Yangyuan, and Ganghuacun at the junction between Wuchang and Qingshan Districts; Guandong in Hongshan District; Caidian in Caidian District; and Zhifang in Jiangxia District. The population in this area can reach up to 3000 people for each grid of 150 m.

The number of low-reaching populations in each street was counted and visualized (Figure 9b). Most of the low-reaching populations are located outside the 60-min walk, which is consistent with the spatial distribution of the low-reachability population. In addition, many low-reaching populations were observed in Hengdian and Yangluo Streets. The main reason is that the two streets are large, and no lake can be found in Hengdian Street. Although Yangluo Street contains several lakes, the road traffic system is underdeveloped. Thus, accessibility to the lake is very poor. Yongfeng, Jiangdi, and Cuiwei Streets in Hanyang District; Dunyang and Zhuankou Streets in Caidian District; Jiufeng Street in Hongshan District; Donghu Scenic Area Street; Peace Street; and other places have the lowest numbers of low-reaching populations, which are less than 350 people. Nearly all of these areas are within a 60-min walk to the lake.

### 4.3. Analysis Results of Urban Lake Service Levels

**Figure 10:**
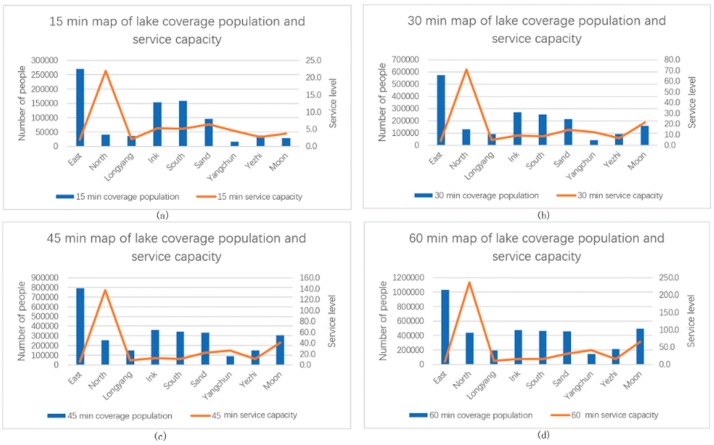
Service level of lakes in the three rings within different service areas: (**a**) 15-min, (**b**) 30-min, (**c**) 45-min, and (**d**) 60-min service areas.

This paper selected the nine major lakes represented by East, Nanhu, and Shahu Lakes in Third Ring Road and 16 major lakes represented by Tangxun, Huangjia, and Jinyin Lakes outside the Third Ring Road as the research objects. The differences in service levels of various lakes were compared and analyzed, as shown in Figure 10 and Table 7.

Among the lakes in the Third Ring Road, East Lake has the largest population, whereas Yangchun Lake has the smallest population. The reason is that East Lake is the largest lake in the Third Ring Road with a large service area, whereas Yangchun Lake has a small area, a small service area, and is located at the edge of the Third Ring Road. Although East Lake has the largest population, the number of lakes per unit length is small. The number of people in the 60-min service area is fewer than eight people. The number of lakes per unit length of the lake is much higher than that of the other lakes. A total of more than 235 people are observed because the area of the North Lake is only 4617 m^2^, which is located in the center of the city. In addition, the surrounding population is extremely dense. Within 60-min of service, six lakes serve a population of 400,000. East and Longyang Lakes have a population of fewer than 10 people per lake length. North and Moon Lakes have a population of more than 30 people per lake length. Two lakes with a population of 10 to 20 and 20 to 30 per unit length of the lake boundary service are also observed.

**Figure 11:**
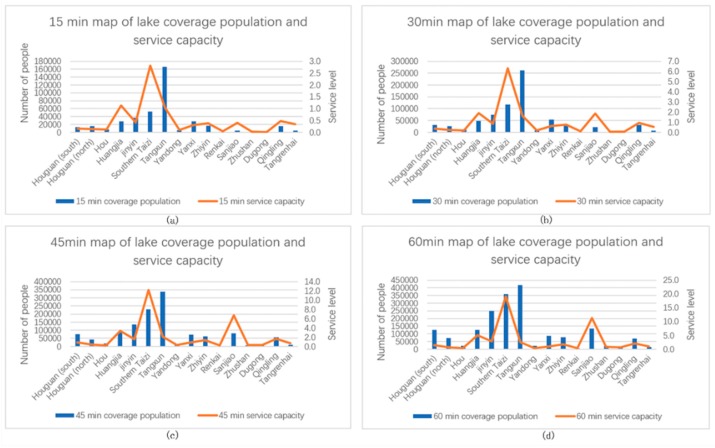
Service level of lakes outside the three rings within different service areas: (**a**) 15-min, (**b**) 30-min, (**c**) 45-min, and (**d**) 60-min service areas.

Among the lakes outside the Third Ring Road, the largest area of Tangsong Lake has the largest service population, followed by the South Taizi Lake, as shown in Figure 11 and Table 8. Six lakes have a service population of fewer than 50,000 within 60-min of the study area. The southern Taizi Lake has the largest number of lakes per unit length of service, which reaches 19 people within 60-min of service. In the 60-min service area of the Sanjiao Lake, the number of lake boundary service population per unit length reaches 11, and the remaining lakes within 60-min of the service range have a population of fewer than five people per unit length.

Comparing the service levels within the gradient service range of the lakes inside and outside the Third Ring Road, we find that regardless of the number of the serving population and number of people serving the lake boundary per unit length, service levels for the lakes inside the Third Ring Road are much higher than those for the lakes outside. The reason is that the population density in the Third Ring Road is larger than that outside, with a more developed road traffic system and larger lake service area

### 4.4. Street Grade Based on Lake Supply and Demand Levels

#### 4.4.1. Classification of Street Grades Based on Lake Supply Levels

**Figure 12:**
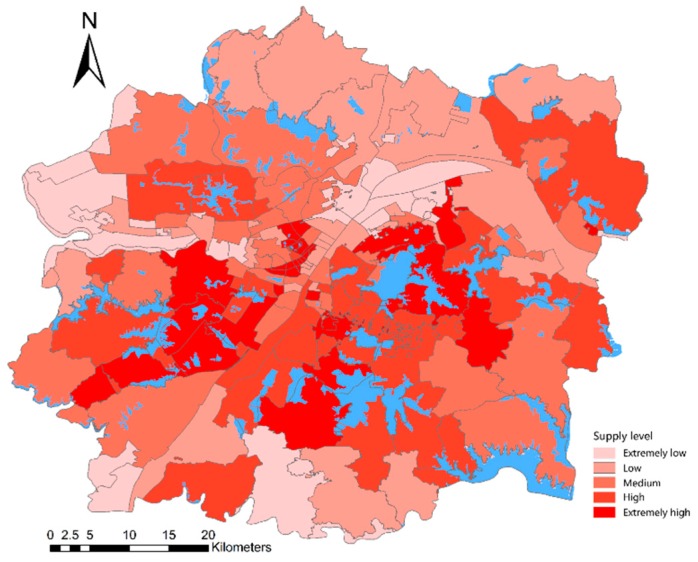
Spatial distribution pattern of lake supply levels.

To characterize the lake supply level of each street, we calculated the proportion of the population of each street within the 60-min service area of the lake to the total population of the street. The streets were divided into five grades, namely, extremely high, high, medium, low, and extremely low supply levels (Figure 12 and Table 9). Streets with extremely high supply levels account for 19.59% of the total number of streets, which are mainly distributed in three areas, namely, East and Yanxi Lakes, Tangsong and Huangjia Lakes, and Houguan and Sanjiao Lakes. These areas are located around the city’s Third Ring Road and the lakes are densely populated. Streets with extremely low supply levels account for 16.22% of the total number of streets, which are mainly located in the vicinity of Tianxing Township, Zoumaling, and the southern edge of the Wuhan urban development zone. Few lakes can be observed in these areas, and the road transportation system here is relatively backward compared with that of the urban center. Streets with medium and above supply levels account for 62.16% of the total number of streets, and most of the streets have a sufficient supply of lakes. Streets with medium and below supply levels are concentrated in the northern, southern, and western parts of the urban development zone, where lake resources are relatively scarce. We focus on these areas.

#### 4.4.2. Street Classification Based on Lake Demand

**Figure 13:**
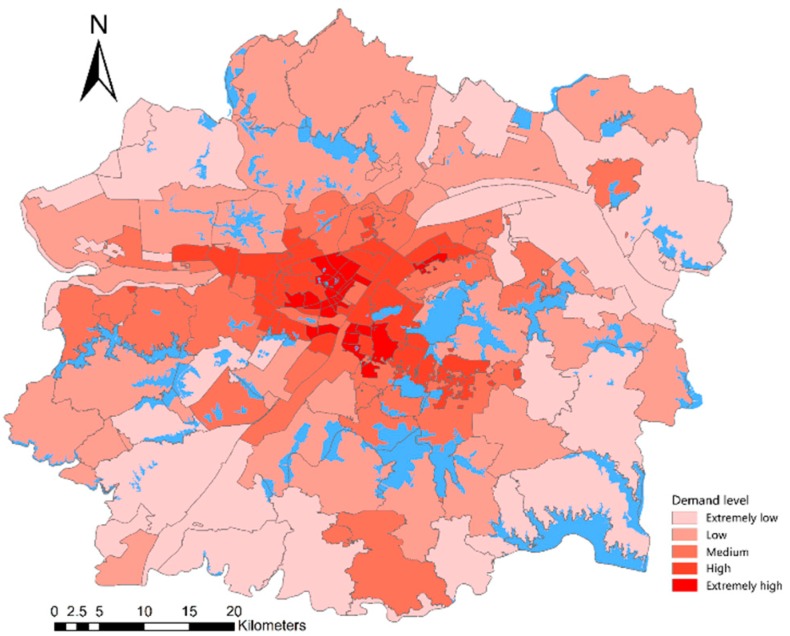
Spatial distribution pattern of lake demand levels.

Data on the density of population groups, such as women, children, the elderly, and non-native populations, and the total population density were used to weight the sum. The results were used to characterize the demand level of lakes in each street, where the streets were divided into five levels, namely, extremely high, high, medium, low, and extremely low demand levels, as shown in Figure 13 and Table 10. Streets with extremely high demand levels account for 20.95% of the total number of streets, which are mainly distributed in Hankou and Wuchang. Both regions are located in the center of the city and have large population densities. Hankou has a dense distribution of business, such as the Wuguang and Jianghan Road business districts. Wuchang has many universities, such as Wuhan University and Huazhong Normal University. Streets with extremely low demand levels account for 18.24% of the total number of streets, which are mainly distributed on the edge of urban development areas, where the population density is relatively small.

In summary, the lake demand level shows a circular distribution pattern, which originates from the center to the periphery. That is, the closer to the city center, the higher the demand level of the lake; the closer to the edge of the urban development area, the lower the lake demand level. In addition, this finding matches the spatial distribution pattern of the urban population.

### 4.5. Macro Assessment of the Fairness of Lakes Distribution

#### 4.5.1. Correlation Analysis of Sequencing Variables

**Figure 14:**
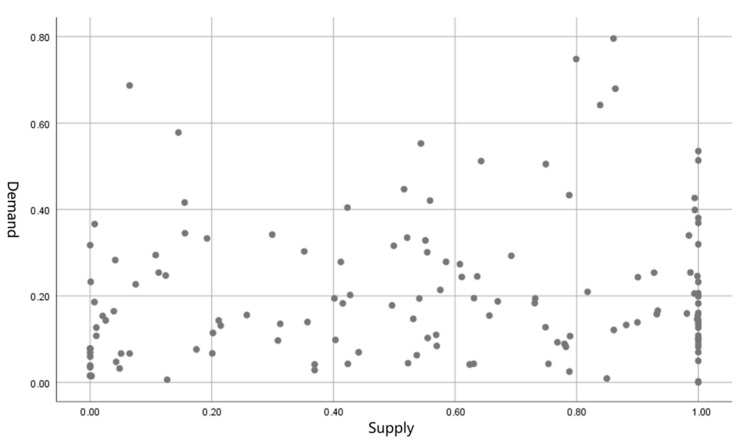
Spot chart of lake supply and demand.

SPSS was used to analyze the supply and demand levels of lakes for each street. Figure 14 and Table 11 provide the results. The Kendall rank correlation coefficient between the supply and demand levels of each street lake is 0.235, whereas the Spearman rank correlation coefficient is 0.329. The statistical values of significance are below the 0.01 level, indicating a significant correlation between the supply and demand levels of the lakes for each street. That is, the supply level of lakes with high lake demand is also high. The supply level of the lake can better match the needs of the residents of the streets, which further indicates that the spatial layout of the lake is reasonable and space fairness is high.

#### 4.5.2. Evaluation of Lake Fairness Based on the Gini Coefficient

This paper used the Gini coefficient to evaluate the balance of the supply and demand in the study area, and concludes that the relationship between supply and demand and the social equity of the income distribution are similar. Referring to the income distribution situation, the smaller the Gini coefficient, the more balanced the supply and demand relationship of urban lakes. The Lorenz curve was graphically represented to indicate the matching of urban lake supply and demand, and can express a certain proportion of lake demand with a certain proportion of lake supply matching.

**Figure 15:**
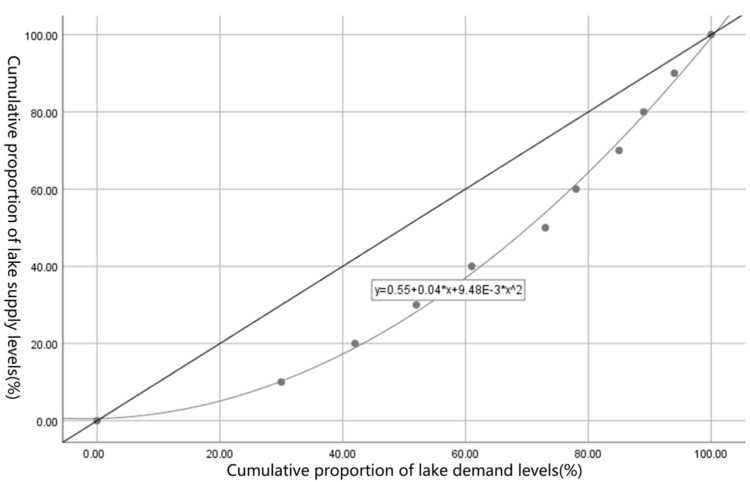
Lorenz curve of the lake supply and demand.

The Gini coefficient of the lake supply and demand in the study area is 0.316, which is in a relatively reasonable stage, indicating that the supply and demand of lakes are relatively balanced. The lake supply index of all the streets in the study range was ranked from low to high, and 10% of the lake demand was used as a section. The proportion of lake supply in each section was calculated and accumulated according to the calculation method of the Lorentz curve (Table 12), and then a Lorentz graph was drawn (Figure 15) that measures the supply and demand relationship of lakes in each street.

It can be seen from the Figure 15 that there is still a certain imbalance in the supply and demand of lakes in the streets of the study area. For the needs of residents with a lesser lake supply, only 10% of the demand for the 1% of the lakes is matched, 20% of the residents have only 3% of the lake supply, and 30% of the residents have only 11% of the lake supply matched; for the needs of residents with more lake supply, 10% of the demand has 18% of the lake supply matched, 20% of the demand has 36% of the lake supply matched, 30% of the demand 51% of the lake supply matched.

### 4.6. Micro Assessment of the Fairness of Lakes’ Distribution Based on Location Entropy

**Figure 16:**
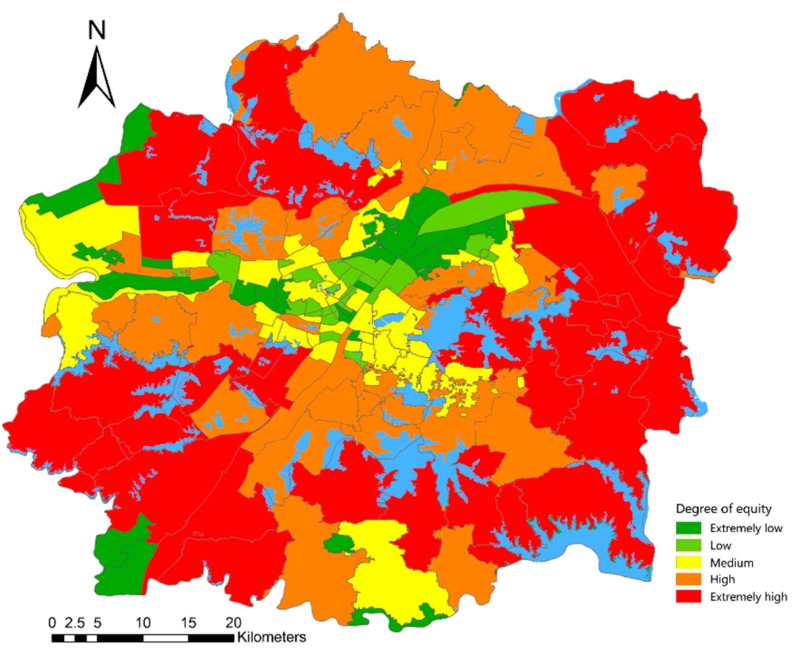
Spatial layout pattern of the fairness of lakes’ distribution.

We used the street location entropy to represent the fairness of lakes’ distribution in space. The fairness of lakes’ distribution was divided into five levels, namely, extremely high, high, medium, low, and extremely low, as shown in Figure 16 and Table 13. The results show that 60 streets have high or high fairness, which accounts for 40.54% of the total number of streets in the study area. These streets are distributed outside the central city. The main reason is that these areas have a small population density; thus, demand is low. The 37 streets are at a medium level, which accounts for 25.00%. These streets are mainly distributed in Wuchang, Jianghan, and Hanyang Districts. The lake resources in these areas are rich, but the population density is very large. Fifty-one streets were considered low or low fairness, which accounts for 34.46%. These streets are mainly distributed in the southeast part of Jiang’an District, northern part of Qingshan District, and southern part of Qiaokou District. Small amounts can be found in the northern part of Wuchang District, southern part of Jianghan District, and southern and northwestern parts of Dongxihu District. Most of these streets are located in the center of the city, are small, and have a high population density.

In summary, the spatial layout of the fairness of lakes’ distribution in the entire urban development zone is roughly in a circular distribution pattern. That is, the closer to the city center, the lower the fairness; the closer to the edge of the urban development zone, the higher the fairness.

## 5. Discussion

Existing research assesses the fairness of urban public space layout, which is frequently based on demographic data, to measure users’ needs [45,46]. For example, the population of each residential area is calculated according to the ratio of the residential area to street population data. The results of the population distribution map are then comprehensively analyzed. The accessibility of the urban public space is investigated to assess the fairness of the urban public space layout. This approach has certain limitations due to the assumption that the population is evenly distributed throughout the residential area. Based on the theory of accessibility and fairness, the current study first used the spatial data of the population based on land cover types to measure the fairness of lakes’ distribution in the study area. Mapping population data according to specific spatial units is more accurate and reliable than traditional methods (population data in administrative divisions). Furthermore, the fairness of lakes’ distribution in the Wuhan city center was quantitatively evaluated from the perspective of supply and demand by studying the population data of different service areas of lakes under specific costs.

The results of this study are intended as a reference for the government in the construction of urban lakeside and blue–green spaces. The spatial distribution of lakes is naturally formed and cannot be changed, so rebuilding lakes is unrealistic. Nonetheless, the government should consider allocating landscape resources in lake areas with unfairness, such as building parks and green spaces, to compensate for the lack of lake landscapes. Constructing lakeside landscapes around lakes with poor service levels and surrounded by road traffic should be strengthened to fully utilize these undeveloped resources for the lake landscape. In addition, several studies demonstrate that the number and area of lakes in the Wuhan urban development zone have been decreasing during a development period of nearly 30 years. This tendency follows the evolution pattern of large lakes as they split into small lakes and disappear. The total lake area has decreased from 376.52 km^2^ in 1987 to 282.43 km^2^ in 2018 [47]. Therefore, the government should intensify lake protection. Otherwise, as the lakes continue to decrease, and urban population continues to increase, the fairness of lakes’ distribution will be further reduced.

Data obtained in this study are limited. Therefore, supplementing the following aspects in the future is necessary.

(1) With remote sensing image data as the basis, this study considered the effect of land cover types on the spatial distribution of the population but ignored the vertical distribution (high-rise buildings). Research is conducted to explore population spatialization methods based on building information [48]. However, building data from field trips are typically suitable for small-scale research. As such, obtaining accurate building height information for the entire urban development area of Wuhan was difficult for our research.

(2) We used Landsat 8 remote sensing images to classify land cover types. The limited resolution of the remote sensing images affects the spatialization results of the population. In addition, modifying the results through manual visual interpretation during visual interpretation introduces the possibility of subjectivity. Therefore, obtaining high-resolution remote sensing images for research is important.

(3) We only studied the accessibility of lakes under walking mode, and only the vulnerable populations were considered as factors affecting the demand of lakes. Economic factors, such as income and living standards of residents, were not considered. In the future, we will study the accessibility of lakes using additional modes of travel and use comprehensive socio-economic data to assess lake equity for specific groups, such as the elderly, women, and children, and incorporate the results into existing lake landscape assets.

## 6. Conclusions

This study obtained an accurate and detailed spatial distribution map of the population by allocating the street population in the Wuhan urban development zone to a 150-m unit grid. Using network analysis, the service scope map of the lake with different travel times at certain costs was obtained. Statistics showed that the service population of the lake within walking distances of 15, 30, 45, and 60 min accounted for 19.44%, 36.28%, 50.47%, and 60.78%, respectively, of the total population in the study area. In addition, the service areas accounted for 8.13%, 16.17%, 23.62%, and 30.60% of the total study area. The proportion of population covered by each gradient service is approximately twice that of the coverage, indicating that people tend to live around lakes in urban development areas.

After comparing the service levels of the lakes inside and outside the Third Ring Road, we found that service levels for the lakes inside the Third Ring Road are much higher than those for the lakes outside, regardless of the number of people served or the number of people serving the lake boundary per unit length. The main reason is that the population density inside the Third Ring Road is larger than that outside, with a more developed road traffic system and larger lake service area. The East Lake in the Third Ring Road has the largest population, whereas Yang Chun Lake has the lowest service population. Six lakes are serving a population of 400,000. The number of lakes per unit length of the East Lake is the lowest, with fewer than eight people within the 60-min service area. Furthermore, the number of lake boundary service population per unit length in North Lake is much higher than that of other lakes, which can reach 235 in 60-min. Outside the Third Ring Road, Tangxun Lake has the largest population, whereas South Taizi Lake has the largest number of lakes per unit length of service, which can reach 19 people within 60-min of service. In addition, six lakes have a service population of less than 50,000 in the 60-min service area and 14 lakes within 60-min of service. The number of lake boundary service population per unit length is less than five people.

After calculating the supply and demand levels of the lakes, we found that streets with extremely low supply levels accounted for 16.22% of the total number of streets, which are mainly distributed in the vicinity of Tianxing Township and Zhoumaling and on the southern edge of the urban development zone. Few lakes are located in these areas, and the road transportation system is relatively backward compared to the urban center. The demand level of lakes showed a circular distribution pattern. The closer to the city center, the higher the lake demand level. Streets with extremely low demand levels account for 18.24% of the total number of streets, which are mainly distributed on the edge of urban development areas where the population density is relatively small.

A significant correlation was observed between the supply and demand levels of the lakes for each street. That is, the supply level of lakes with high lake demand is also high. The supply level of the lake can match the needs of the residents of the streets, so the spatial layout of the lake is reasonable and space fairness is high. According to the overlay analysis results, the spatial layout of the fairness of lakes distribution in the entire urban development zone follows a circular distribution. In other words, the closer to the city center, the lower the fairness; the closer to the edge of the urban development zone, the higher the fairness. Fifty-one streets have low or low fairness, accounting for 34.46%. These streets are mainly distributed in the southeast part of Jiang’an District, northern part of Qingshan District, and southern part of Qiaokou District. Small amounts were observed in the northern part of Wuchang District, southern part of Jianghan District, and southern and northwestern parts of Dongxihu District. The majority of these streets are located in the center of the city with a small area and a dense population.

This study evaluated the fairness of lakes in the Wuhan urban development zone and obtained a detailed spatial layout of the fairness of lakes’ distribution. It identified areas with poor fairness of lake distribution, explained the reasons, and put forward relevant suggestions for urban construction. Along with relevant research in cities, this study provides valuable references.

## Figures and Tables

**Table 1:** Streets in the study area.

District	Street Name	Number of Streets
Caidian	Fenghuangshan Yuxianzhen Zhangwan Caidian Sheshan Tunkou Houguanhu Changfu Tunyang Junshan Daji	11
Dongxihu	Dongshan Cihui Changqing Zoumaling Changqinghuayuan Jinyinhu Jiangjunlu Wujiashan Baiquan Jinghe Xingouzhen	11
Hannan	Shamao Dongjing	2
Hanyang	Yingwujie Yuehu Jianqiao Qingchuan Jianghanerqiao Sixin Wulidun Jiangdi Cuiwei Zhoutou Qinduankou Yongfeng	12
Hongshan	Shizishan Liyuan Jiufeng Tianxingxiang Donghu Zhuodaoquan Hongshan Guanshan Qingtanhu Heping Bajifu Huashan Qingling Luonan Zuoling Zhangjiawan Guandong	17
Huangpi	Tianhe Panlongcheng Wuhu Niekou Sanliqiao Hengdian Qijiawan	7
Jiangan	Laodong Qiuchang Siwei Dazhi Yongqing Erqi Yiyuan Huaqiao Xincun Chezhan Danshuichi Taibei kanjiaji Houhu Xima Baibuting Tazihu	17
Jianghan	Manchun Beihu Tangjiadun Jianghan Minzu Qianjin Hualou Wansong Minquan Changqing Hanxing Xinhua Shuita Minyi	14
Jiangxia	Wulongquan Canglongdao Wulijie Liufang Liangzihu Miaoshan Binhu Jinkou Zhifang Jingang Zhengdian Fozuling Baoxie Daqiaoxinqu	14
Qiaokou	Ronghua Hanjiadun Hanzhong Liujiaoting Gutian Hanzheng Changfeng Baofeng Hanshuiqiao Zongguan Yijia	11
Qingshan	Honggangcheng Baiyushan Yejin Qingshanzhen Gangduhuayuan Wugangchangqu Xingouqiao Gongrencun Ganghuacun Hongweilu Changqian Wudong	12
Wuchang	Huanghelou Nanhu Shuiguohu Shouyilu Ziyang Zhongnanlu Luojiashan Donghu Zhonghualu Baishazhou Shidong Jiyuqiao Yangyuan Liangdaojie Xujiapeng	15
Xinzhou	Cangqu Yangluo Xinzhou Zhangduhu Shuangliu	5

**Table 2:** Main lakes in the study area (key protected lakes within the scope of Wuhan lake protection regulations).

Administrative Region	Lakes
Wuchang District	Sand Ziyang Fruit Simei East
Hanyang District	Moon Lotus Ink Longyang
Qingshan District	North
Hongshan District	Qingling Huangjia South Ye Yezhi Yangchun Tangxun Bamboo Qingtan Yanxi Yandong
Caidian District	West Small-Zha Guanlian Houguan Zhiyin Southern-Taizi Northern-Taizi Sanjiao Lanni Tang Wanjia
Jaingxia District	Niushan An
Jiangan District	Wanzi and Tazi Lekes
Jianghan District	Machine-Pond North Lingjiao
Qiaokou District	Zhangbi
Huangpi District	Hou Tongjia Majia Maijia Shizi Renkai Xinjiao Panlong Chang Zhangzhou
Xinzhou District	Wu Zhujia Taoshu Taojia Chaibo

**Table 3:** Division of the Gini coefficient fractional segment.

Fractional Segment	Significance
<0.2	Absolute average income
0.2–0.3	Average income
0.3–0.4	Relatively reasonable income
0.4–0.5	Large income gap
≥0.5	Huge income gap

**Table 4:** Spearman correlation analysis between population and land cover types.

Land Cover Type	Water	Wood Land	Farm Land	Bare Land	Road	Residential Building	Factory Building	Other Paving	Unclassified Land
Correlation coefficient	0.025	0.023	−0.051	0.011	0.305 **	0.397	0.041	0.509 **	−0.315 **
Significance level	0.376	0.388	0.261	0.444	0.000	0.000	0.303	0.000	0.000

** Correlation is significant at 0.01 level.

**Table 5:** Comparison of OLS and GWR model results.

	Model	GWR	OLS
	VIF	5.21 (Road) 4.32 (Residential land) 1.55 (Other paving)	
Model parameter	AIC	3815.341	3883.771
	R^2^	0.664	0.190
	R^2^ adjusted	0.559	0.180
	MI	0.002	0.135

**Table 6:** Population and area statistics in the lake service area.

Coverage Area (min)	Service Population (People)	Metropolitan Population (People)	Percentage (%)	Service Area (km^2^)	Metropolitan Area (km^2^)	Percentage (%)
15	1,615,420	8,308,841	19.44	267.78	3294.34	8.13
30	3,014,743	8,308,841	36.28	532.53	3294.34	16.17
45	4,193,591	8,308,841	50.47	777.98	3294.34	23.62
60	5,050,275	8,308,841	60.78	1007.97	3294.34	30.60

**Table 7:** Service level of lakes in the three rings within different service areas.

Lake	15-min Coverage Population (People)	15-min Service Capacity (People/m)	30-min Coverage Population (People)	30-min Service Capacity (People/m)	45-min Coverage Population (People)	45-min Service Capacity (People/m)	60-min Coverage Population (People)	60-min Service Capacity (People/m)	Area (m^2^)	Perimeter (m)
East	270,225	2.0	572,922	4.2	793,051	5.8	1,032,509	7.6	32,773,254	135,589
North	40,754	21.9	131,224	70.6	255,549	137.4	438,225	235.6	96,300	1860
Longyang	35,979	2.0	90,746	5.1	148,689	8.3	193,491	10.8	1,329,300	17,880
Ink	154,478	5.2	271,596	9.2	360,325	12.2	477,751	16.1	3,017,507	29,621
South	158,426	5.2	250,683	8.2	341,263	11.2	464,703	15.2	7,391,272	30,595
Sand	95,716	6.4	212,406	14.3	331,736	22.3	457,504	30.7	2,619,000	14,880
Yangchun	15,633	4.5	42,229	12.1	90,083	25.9	143,037	41.1	297,900	3480
Yezhi	38,061	2.7	92,810	6.6	150,840	10.8	213,661	15.2	1,523,061	14,030
Moon	28,300	3.8	158,921	21.2	306,247	40.9	490,518	65.5	575,012	7493

**Table 8:** Service level of lakes outside the three rings within different service areas.

Lake	15-min Coverage Population (People)	15-min Service Capacity (People/m)	30-min Coverage Population (People)	30-min Service Capacity (People/m)	45-min Coverage Population (People)	45-min Service Capacity (People/m)	60-min Coverage Population (People)	60-min Service Capacity (People/m)	Area (m^2^)	Perimeter (m)
Houguan (south)	13,742	0.2	32,324	0.4	76,907	0.9	126,675	1.4	13,283,325	87,651
Houguan (north)	15,254	0.1	25,262	0.2	43,562	0.4	73,615	0.7	12,041,513	112,248
Hou	8198	0.1	13,087	0.2	18,107	0.3	22,290	0.3	15,390,729	70,334
Huangjia	28,461	1.1	48,340	1.9	86,780	3.4	127,036	5.0	7,256,347	25,375
Jinyin	37,066	0.4	74,590	0.8	135,072	1.5	249,437	2.8	8,109,849	88,360
Southern Taizi	52,501	2.8	117,874	6.3	228,546	12.2	358,287	19.1	2,643,300	18,720
Tangxun	16,6236	1.0	261,963	1.7	337,915	2.1	417,903	2.6	42,682,917	158,565
Yandong	5384	0.1	9798	0.2	14,939	0.3	21,086	0.4	7,751,278	51,655
Yanxi	27,809	0.3	53,905	0.6	74,374	0.8	86,019	1.0	13,289,642	88,498
Zhiyin	16,691	0.4	32,845	0.7	61,739	1.4	77,638	1.8	6,355,800	44,220
Renkai	534	0.0	980	0.1	2482	0.2	4770	0.4	1,348,931	11,675
Sanjiao	4695	0.4	22,384	1.9	81,068	6.8	135,723	11.3	2,095,565	11,989
Zhushan	565	0.0	2151	0.1	8780	0.3	21,780	0.8	3,438,243	263,96
Dugong	75	0.0	1169	0.0	6552	0.3	15,743	0.6	2,016,026	24,412
Qingling	15,950	0.5	31,301	0.9	55,950	1.7	70,812	2.1	5,866,225	33,678
Tangren hai	4376	0.3	6896	0.5	9225	0.7	12,486	1.0	984,600	12,600

**Table 9:** Classification of lake supply levels.

Supply Level	Street Name	Number of Streets and Proportion
0.0000–0.1267 Extremely low	Shamao Wulongquan Dongshan Cihui Qingshanzhen Shidong Liangzihu Canjiaji Dongjing Hongweilu Xingouzhen Danshuichi Gutian Tianxingxiang Baibuting Hanjiadun Zhangwan Zoumaling Zhengdian Gangduhuayuan Honggangcheng Xincun Gongrencun Zhangduhu	24 16.22%
0.1268–0.3692 Low	Wulijie Zhifang Yuxianzhen Hengdian Jingangxinqu Qijiawan Zongguan Xingouqiao Hualou Caidian Yangyuan Yijia Yongqing Jianghan Sanliqiao Minzu Minquan Siwei Bajifu Niekou Ganghuacun Yiyuan Changqing Wuhu Qingtanhu Changqian Jianqiao Tianhe Cangqu Zhonghualu Changqingjie Erqi	32 21.62%
0.3693–0.6112 Medium	Houhu Donghu Shuangliu Qingchuan Panlongcheng Laodong Changfeng Wansong Baiquan Zhoutou Sheshan Beihu Yejin Junshan Jianghanerqiao Wujiashan Baoxie Liyuan Baofeng Hanshuiqiao Shouyilu Yuehu Tazihu Yingwu Jiyuqiao Hanxing Hougonghu Jiangjunlu Fozuling Liufang Ziyang	31 20.95%
0.6113–0.8496 High	Baishazhou Fenghuangshan Qingling Jinyinhu Zuoling Guandong Yangluo Zhuodaoquan Huashan Miaoshan Zhangjiawan Xujiapeng Wudong Zhongnanlu Shizishan Luonan Sixin Guanshan Shuigouhu Canglongdao Jinghe Jinkou Liangdaojie Luojiashan Baiyushan Binhu Xinhua Wulidun Qinduankou Changqinghuayuan Daji Tunkou	32 21.62%
0.8497–1.0000 Extremely high	Hongshan Tangjiadun Beihu Xima Taibei Ronghua Manchun Huanghelou Manhu Qiuchang Hanzhong Qianjin Dazhi Liujiaoting Jiufeng Xinzhou Wugangchangqu Chezhan Tunyang Hanzheng Heping Jiangdi Cuiwei Shuita Minyi Tunkou Daqiaoxinqu Yongfeng Huaqiao	29 19.59%

**Table 10:** Classification of lake demand levels.

Demand Level	Street Name	Number of Streets and Proportion
0.0013–0.0527 Extremely low	Liangzihu Wugangchangqu Jingangxinqu Baiquan Tianxingxiang Zhangduhu Liufang Tunyang Dongjing Binhu Wulongquan Yuxian Dongshan Yangluo Junshan Bajifu Zhengdian Shuangliu Sixin Baoxie Zhangwan Sanliqiao Wulijie Jiufeng Jinkou Qingtanhu Xingouzhen	27 18.24%
0.0528–0.1252 Low	Miaoshan Qijiashan Cangqu Daji Panlongcheng Zoumaling Sheshan Tianhe Wuhu Huashan Hengdian Donghu Daqiaoxinhu Jinghe Niekou Zuoling Cihui Beigong Tunkou Canglongdao Fozuling Shamao Zhangjiawan Jiangdi Jinyinhu Linkong Kanjiaji	28 18.92%
0.1253–0.2161 Medium	Wudong Shidong Changqing Jiangjunlu Guandong Yongfeng Tazihu Fenghuangshan Hougonghu Zhifang Yuehu Gongrencun Baishanzhou Qingshanzhen Baiyushan Shizishan Hongshan Zhoutou Honggangcheng Danshuichi Heping Yangluo Changqian Caidian Hualou Liyuan Gutian Qingchuan Yiyuan Houhu	30 20.27%
0.2162–0.4196 High	Yingwu Luonan Yangyuan Jiyuqiao Wansong Yongqing Luojiashan Shuiguohu Guanshan Erqi Changqinghuayuan Xincun Xujiapeng Hongweilu Wulidun Changfeng Wujiashan Jianghan Qinduankou Zongguan Jianghanerqiao Hanzhong Baibuting Hanxing Changqing Minzu Hanjiadun Zhuodaoquan Shouyilu Xingouqiao Yijia Yejin	32 21.62%
0.4197–1.0000 Extremely high	Jianqiao Siwei Zhongnanlu Hanshuiqiao Nanhu Laodong Ziyang Baofeng Gangduhuayuan Cuiwei Beihu Xinhua Huanghelou Liangdao Xima Tangjiadun Zhonghualu Taibei Ganghuacun Qiuchang Huaqiao Shuita Chezhan Liujiaoting Ronghua Minquan Dazhi Hanzheng Manchun Minyi Qianjin	31 20.95%

**Table 11:** Correlation analysis of sequential variables of supply and demand in lakes.

Supply Level	**Demand Level**		
	Relativity test	Kendall’s tau_b	Spearman’s rho
	Correlation coefficient	0.235 **	0.329 **
	Significance level	0.000	0.000

** Correlation is significant at the 0.01 level.

**Table 12:** The cumulative proportion of the lake demand and supply levels.

Cumulative Proportion of Lake Demand Levels (%)	Cumulative Proportion of Lake Supply Levels (%)
10	1
20	3
30	11
40	18
50	28
60	39
70	48
80	63
90	82
100	100

**Table 13:** Classification of the fairness of lakes’ distribution based on location entropy.

Degree of Equity	Street Name	Number of Streets and Proportion
0.00–0.30 Extremely low	Shamao Wulongquan Dongshan Cihui Qingshanzhen Shidongjie Liangzihu Kanjiaji Dongjing Hongweilu Xingouzhen Danshuichi Hanjiadun Baibuting Gangduhuayuan Xincun Minquan Xingouzhen Ganghuacun Zongguan Siwei Minzu Zhonghualu Honggangcheng Qianjin Gutian	26 17.57%
0.31–0.66 Low	Jianghan Minyi Manchun Yijia Hanzheng Dazhi Yangyuan Erqi Yongqing Ronghua Liujiaoting Changqing Jianqiao Gongrencun Chezhan Shuita Huaqiao Baofeng Qiuchang Hualou Tianxingxiang Hanshuiqiao Ziyang Taibei Laodong	25 16.89%
0.67–2.00 Medium	Zoumaling Shouyilu Tangjiadun Yejin Liangdao Caidian Xima Zhifang Xinhua Jianghanerqiao Huanghelou Hanxing Zhongnanlu Zhuodaoquan Beihu Cuiwei Zhangwan Wujiashan Nanhu Yiyuan Wansong Hanzhong Houhu Xujiapeng Qinduankou Jiyuqiao Yingwu Wulidun Wugangchangqu Shuiguohu Qingchuan Changqinghuayuan Guanshan Changqian Luojiashan Luonan Changfeng	37 25.00%
2.01–19.74 High	Zhengdian Liyuan Yangluo Zhoutou Hengdian Changqing Wulijie Yuehu Heping Tunkou Shizishan Baishazhou Tazihu Qijiawan Fenghuangshan Jiangjunlu Hongshan Zhangduhu Yuxianzhen Baiyushan Duandong Yongfeng Wudong Jinyinhu Sanliqiao Zhangjiawan Wuhu Donghu Fozuling Qingling Hougonghu Niekou	32 21.62%
≥19.75 Extremely high	Tianhe Bajifu Jiangdi Beigong Cangqu Qingtan Dunkou Canglongdao Zuoling Panlongcheng Sheshan Jingang Jinghe Huashan Donghu Miaoshan Baoxie Daji Junshan Jinkou Jiufeng Sixin Liufang Yangluo Binhu Baiquan Tunyang Shuangliu	28 18.92%
